# Remediation of Water Using a Nanofabricated Cellulose Membrane Embedded with Silver Nanoparticles

**DOI:** 10.3390/membranes12111035

**Published:** 2022-10-24

**Authors:** Salma Shad, Iseult Lynch, Syed Waqar Hussain Shah, Nadia Bashir

**Affiliations:** 1 Department of Chemistry, Faculty of Natural Science, The University of Haripur, Haripur 22620, Pakistan; 2 Department of Chemistry, Hazara University, Mansehra 21300, Pakistan; 3 School of Geography, Earth and Environmental Sciences, University of Birmingham, Edgbaston, Birmingham B15 2TT, UK

**Keywords:** pesticide contamination, nanoparticle-enhanced polymeric membrane, adsorption, remediation, agricultural run-off

## Abstract

The removal of pesticide pollution is imperative, because of their high environmental load and persistence, and their potential for bioaccumulation in, and toxicity to the environment. Most pesticides are found to be toxic even at trace levels. AgNPs can be effectively used for the adsorption of pesticides, and the incorporation of the AgNPs onto a support polymeric membrane enhances their effectiveness and reduces the potential unwanted consequences of intentionally adding free nanoparticles to the environment. Here, silver nanoparticles (AgNPs) were synthesized using a reliable, eco-friendly, and one-step “green” method, by reacting *Mentha Piperita* (mint) extract with AgNO_3_ aqueous solution at 60 °C in a microwave. The resulting high surface area nanoparticles are both economic and effective environmental remediation agents, playing a promising role in the elimination of aquatic pesticide pollution. Embedding the nanoparticles into a cellulose membrane at a low concentration (0.1 g) of AgNPs was shown to result in effectively adsorption of representative pesticides (Cypermethrin, Paraquat, and Cartap) within 60 min, while increasing the concentration of nanoparticles incorporated into the membrane further enhanced the removal of the exemplar pesticides from water. The high adsorption capacity makes the cellulose-AgNPs membrane an excellent substrate for the remediation of pesticide-polluted water.

## 1. Introduction

Pesticides are well-known environmental pollutants, with many possessing mutagenic properties leading to induction of mutations, chromosomal alterations or DNA damage, adversely affecting ecosystems and humans. According to the World Health Organization (WHO), based on a systematic review of reporting data from 141 countries from 157 articles and data from the WHO Mortality Database, approximately 740,000 annual cases of UAPP were reported with about 10% of these resulting in fatalities. Scaling this up, leads to an estimate of 385 million cases of pesticide poisoning annually world-wide [1]. Occupational exposures and work-related contact with pesticides account for 70% of the mortalities, and chronic exposure is associated with a group of syndromes, including numerous tumors and nervous system disorders [2]. Insecticides mostly target the nervous system including the acetylcholine receptor (e.g., a target for neonicotinoids) and acetylcholinesterase, an enzyme which plays an important role in the mediation of nerve impulses [3]. 

Despite their purpose in killing pests, insecticides and herbicides have been allowed for restricted use to control pests or weeds in agriculture, with restrictions including the amounts, rates of applications and crops on which they can be applied, and as data accumulates on their hazards some are banned outright [4]. Besides agriculture, pesticide control is essential in dwellings, laboratories, and food processing installations. Significantly, these chemicals are often human carcinogens, with exposure via ingestion, absorption through the skin, or inhalation, and their long-term exposure may prove fatal [5]. Hence, minimizing pesticide usage and eliminating their excess run-off in the environment is desirable. However, the high binding affinity of soil serves as a storehouse of pesticides and the metabolites [6,7]. Water resources are susceptible to pesticide contamination because of the close interconnection of soil and water bodies. The assimilation of contaminated water can mimic the human body’s hormones that reduce immunity and prevent chronic and reproductive issues [8].

**Figure 1:**
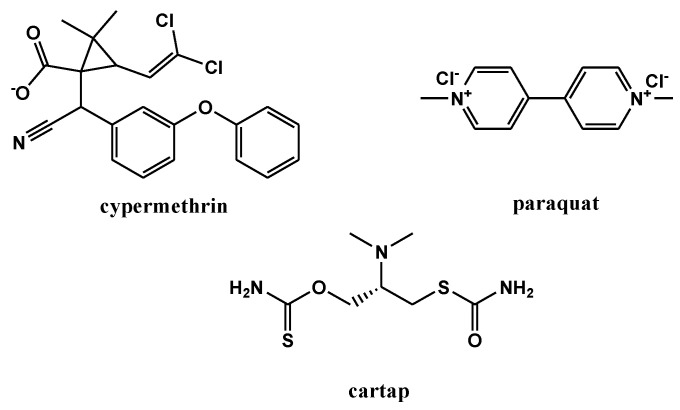
The chemical structures of the tested pesticides—Cypermethrin (CP), Paraquat (PQ), and Cartap hydrochloride (CA).

Cypermethrin (CP), a synthetic pyrethroid, and Cartap (CA) a thiocarbamate insecticide inhibit the nervous system of insects, causing paralysis and eventual death [8,9]. CA has been identified as a toxic pollutant, inducing endocrine disruption, neurotoxicity, and can also affect carbon and nitrogen fixation of cyanobacteria [9]. Highly reactive free radicals are generated during the utilization of Paraquat (PQ), which leads to lipid peroxidation, membrane damage and cell death in plants and mammals. PQ is mainly absorbed orally and via inhalation by animals and becomes highly concentrated in kidneys and lungs [10]. The structures of the herbicides/insecticides used in the present study are provided in Figure 1. Based on the toxicological effects of these pesticides, their widespread use globally, and their range of physicochemical properties (see Table 1), the assessment of their removal from water using sustainable and low-cost approaches is of great interest.

The development of low-cost solutions for the removal of pesticides from aquatic systems is an active research field, with a range of techniques suggested for removal of pesticide contaminants including hydrogen peroxides, ultrasonic waves, hybrid processes, bioremediation, photocatalytic degradation, adsorption, membrane separation, bio-purification systems, composite materials, ion exchange resins, carbon nanotubes, graphene, and nanocrystalline metal oxides [11]. For example, nanoparticles (NPs) formed by noble metals such as silver can be harnessed for remediation of agricultural soils and contaminated water bodies, owing to their ability to bind and degrade pesticides [12]. NPs have gained immense interest for use in biomedicine, drug delivery, optoelectronics, catalysis, optics, and environmental protection [13,14]. Several physio-chemical techniques have been extensively employed for AgNPs synthesis such as microwave, sonochemical, hydrothermal, wet chemical, sol-gel, etc., but have some disadvantages such as the usage of high beam energy, toxic wastes, high costs, and toxic byproducts production, causing severe environmental contamination [15,16]. To overcome these limitations, development of eco-friendly techniques is needed. Green or sustainable chemistry is the chemical philosophy that encourages the design of products and processes which eliminate the use or generation of hazardous substances [17]. One such approach is the exploitation of biological molecules for the synthesis of NPs using biomolecules as reducing and capping agents for NPs [16,18]. Through the green approach, NPs generally have a broad size distribution, because of the presence of several reducing species and a variety of capping agents in an extract, which necessitate control and optimization [16,17,18,19,20]. Another green approach involves the use of microwave-assisted reactions (MAR), which supports the biosynthesis of NPs by providing the high temperature needed for the nucleation process, thereby enhancing the reaction kinetics and reducing the reaction times and associated energy costs [21,22,23]. NPs produced through microwave heating have excellent crystallinity as microwaves provide great control over the morphology and shape of the nanostructured particles [21].

AgNPs synthesized by the green method have been successfully employed in numerous pharmaceutical and biomedical fields due to their high chemical stability [24,25]. AgNPs absorb and scatter light with extraordinary efficiency due to localized Plasmon, with the interaction of electromagnetic waves leading to localized Plasmon resonance. Furthermore, the absorption and scattering properties of NPs could be altered by controlling the particle size, crystal shape, and refractive index [26,27]. NPs provide a highly effective solution to clean up the environment and support remediation by acting as a chemical reductant. Their capacity to adsorb biomolecules and interact with biological receptors means that NPs can reach sub-cellular locations, leading to potentially higher localized concentrations of ions once those particles start to dissolve or degrade in situ [28]. AgNPs are used in several consumer products, whereby significant amounts go down the drain and are eventually released into the sewer system and reach wastewater treatment plants. Through waste treatment, NPs are converted into free silver ions, agglomerate, or interact and complex with ligands such as sulfides present in sludge and effluent [29,30,31]. These environmental transformations can affect the properties of the suspended NPs that result in changes in their aggregation or oxidation states, precipitation of secondary phases, or sorption of organic species, resulting in the formation of colloidal solutions that are potentially toxic [32]. Thus safe use of NPs for environmental applications, such as adsorption and removal of pesticides due to enhanced reactivity, many edges, and high-energy surface defects [33], requires understanding of their environmental transformations.

Composite membranes functionalized with NPs have been shown to remove contaminants from water due to their binding ability through ultra-, micro-and nano-filtration, and reverse osmosis [34,35,36]. Fabrication of membranes that entrap the NPs reduces the risks of NPs addition into and transformation by the environment, and can also prevent biomolecule fouling of the membrane due to the accumulation of salts, colloids, and macromolecules [37]. The antimicrobial properties of AgNPs can reduce the membrane biofouling, thus providing a win-win low-cost solution [38]. The dense nano-pores of microfiltration (MF) membranes, with a microporous structure such as cellulose filter paper, have drawn increasing interest as a means to remove colloids and suspended solids effectively from drinking water sources [39,40]. With efficient antibacterial activities, AgNPs are actively used in MF membrane preparation with noncovalent interactions between NPs and the membranes [41,42,43,44,45,46,47,48]. In many cases, the leaching of AgNPs into treated drinking water will be inevitable, which is a potential threat to both humans and the environment [49,50]. The fabrication of AgNPs into cellulose acetate membranes makes the surface more hydrophilic by allowing an increase in the flux that permeates the membrane [42]. A polymer such as cellulose acetate is preferred in water remediation technologies because of its net negative surface charge that allows control over biofouling. Besides, cellulose acetate membranes are biodegradable, resistant to chlorine, hydrophilic, renewable, and inexpensive [41,42,43,44,45,46,47,48].

In the present work, AgNPs were synthesized by reducing Ag ions in an aqueous solution of mint extract with the precursor salt of silver nitrate (AgNO_3_). With the presence of chemical constituents (terpenoids, flavonoids, phenolic acid, polyphenols, proteins, sugar, etc.) in the plant extract, reduction occurs. The multifunctional AgNPs were characterized and fabricated into the composite cellulose acetate membrane. The efficiency of the AgNPs and composite membrane for the removal of organic pollutants were studied. The process was optimized to eliminate any inherent shortcomings and presents the first evidence of the effectiveness of these low-cost sustainable AgNPs-cellulose acetate membranes at removing pesticides from drinking water.

## 2. Materials and Methods

### 2.1. Materials

Silver nitrate (AgNO_3_) and cellulose acetate were purchased from Sigma-Aldrich (Merck KGaA, Darmstadt, Germany). Herbicides (CP & PQ) and an insecticide, cellulose acetate (CA) was purchased from Merck (Sigma-Aldrich, Darmstadt, Germany). Methanol, ethanol (99% purity, Merck, Sigma-Aldrich) and acetone (100% pure) used in the work were of high purity and analytical grade. All chemicals were utilized as received. 

### 2.2. Green Synthesis of Silver Nanoparticles 

**Figure 2:**
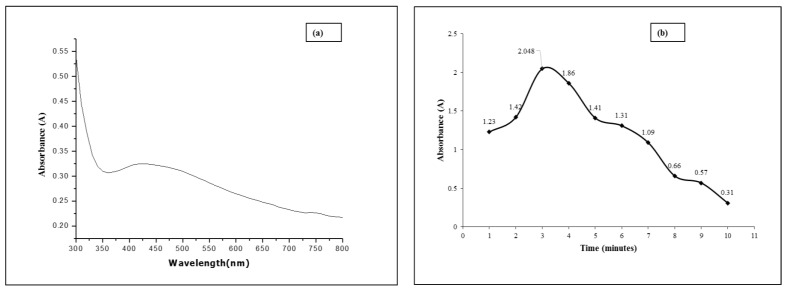
(**a**) The surface plasmon absorbance spectrum of the 20 nm AgNPs produced using the optimized conditions: (**b**) Effect of exposure time on the synthesis of nanoparticles using microwave (MW) irradiation determined at 425 nm.

The plant extract was prepared according to the procedure reported elsewhere [21]. Briefly, twenty milliliters (20 mL) of filtrate of *Mentha* leaf extract was mixed with 5 mM silver nitrate solution in a 1:2 *v/v* ratio at 60 °C and heated using a microwave {INPUT: 230–240 V~50 Hz 1200 W (MICRO), OUTPUT: 700 W 2450 MHz (Boss international company)} set at 48 °C. A visible brown color change occurred within 2 min, which indicated the formation of NPs. Un-aggregated AgNPs gave a yellow color to the solution. The solution was centrifuged for 20 min at 5000 rpm and decanted at room temperature. The residual supernatant was removed by using a pipette, and the solid pellet was washed with deionized water. Further purification was carried out by centrifugation at 15,000 rpm for 20 min. This process was repeated at least three times, and the lower pellet was collected and dried at 60 °C in an oven. The dried pellet was then transferred to an airtight vial and kept in the dark for further use. The synthesis time was optimized from 1 to 10 min and analysis was done by using a UV/Visible spectrophotometer. At the heating time duration of 2 min, higher absorbance and better productivity of nanoparticles were optimized as shown in Figure 2b.

### 2.3. Fabrication of AgNPs and Composite Membrane

2.5 g of cellulose acetate was dissolved in 50 mL of acetone and stirred until complete dissolution. To this solution, the selected amount of AgNPs was added with stirring to form a homogeneous mixture. To be comparable with the free NP adsorption studies, we added 0.1 mg AgNPs to the cellulose acetate solution. The viscous solution thus obtained was cast onto a dry glass plate for film formation. The thin film was air-dried for 30 s and then immersed in 100% pure ethanol until needed. A similar method was repeated for the preparation of a blank membrane without the addition of AgNPs. Later, a 2 cm piece of the fabricated membrane was cut and used for the removal of contaminants [21].

### 2.4. Characterization of Biosynthesized Nanoparticles and Composite Membrane

All characterization was performed at the School of Geography, Earth, and Environmental Sciences, University of Birmingham. The reduction of silver ions by plant extract was followed by recording time-dependent absorbance using a Perkin Elmer Lambda-25 UV-Visible Spectrophotometer. The size distribution was analyzed by nanoparticle tracking analysis (NTA) using a Nano Sight (LM-20). Elemental analysis was done through energy dispersive x-ray (EDX) spectroscopy. Zeta potential, particle size, and the stability of AgNPs were determined through dynamic light scattering, DLS (Zeta sizer Nano series). Silver nanoparticles were studied on carbon-coated copper grids and analyzed using a JEM 1400 Transmission Electron Microscope (TEM) operating at a voltage of 80 kV. About 500 nanoparticles were studied by using Image J software to measure the size of the AgNPs. X-ray diffraction (XRD) analysis was performed by X-ray Diffractometer Model Analytical X-pert Pro to study the structure and shape of NPs. Atomic force microscopy (AFM) was harnessed for analysis of the AgNPs-cellulose acetate composite membrane thickness and surface roughness, as well as to monitor the removal of pesticide/insecticide by binding to the membrane surface.

### 2.5. Efficiency of Biogenic AgNPs for Adsorption of Insecticides and Herbicides from a Contaminated Water Sample

The adsorption/removal of pesticides from the water samples was studied using the AgNPs added directly to the water sample and using the AgNPs-cellulose acetate membrane with a comparable total amount of AgNPs. Pesticide solutions of 1 ppm, 5 ppm, and 10 ppm concentrations were prepared.

(1) Treatment with Silver Nanoparticles 

A fixed amount (0.1 mg) of the dried AgNPs was added to each (1 ppm, 5 ppm & 10 ppm) herbicide/insecticide solutions. The pH and time-dependent absorbance (A) were studied at the maximum absorbance wavelength for each analyte (λ = 617 nm with A = 0.031 for CP, λ = 320 nm with A = 0.037 for CA, and λ = 408 nm with A = 0.191 for PQ, respectively) were recorded. The effect of variation of the initial pesticide concentration (at a constant AgNPs concentration) was also determined. 

(2) Treatment with Composite Membrane

The nanofabricated composite membrane was cut into small pieces (2 cm) and dipped into the pesticide solutions (CP, CA, and PQ) at room temperature. The time-dependent absorbance was measured every 120 s (2 min) over 60 min to follow the entrapment/mineralization of the contaminant onto the AgNPs-loaded membrane. The absorbance was recorded at 617 nm, 320 nm, and 408 nm for CP, CA, and PQ, respectively. The adsorption capacity of the nanocomposite membrane was determined using Equation (1) as follows:

(1) $$q_{e}=\frac{C_{0}-C_{e}}{M}\times \;V$$

where C_e_ and C_0_ are the pesticide concentrations at equilibrium and initial conditions (mg L^−1^), respectively. The parameter of M is the mass of AgNPs (g) and V (L) is the volume of the solution. The percentage of pesticide adsorption (% adsorption) was calculated by Equation (2) as follows:

(2) $$R=(1-\frac{C_{0}}{C_{e}})\times 100$$

All adsorption experiments were conducted in triplicate, and the mean and average values of the three runs are reported. Adsorption was evaluated as a function of time at varied CP concentrations (1 ppm, 5 ppm & 10 ppm), with an adsorbent dose of 0.1 g AgNPs, and a volume of CP solution of 100 mL, based on the amount of CP remaining in solution over time; thus, a decrease in CP concentration correlates with CP adsorption on the AgNPs surface and its removal from the water.

## 3. Results

### 3.1. UV-Visible Analysis of the Synthesized Nanoparticles

The UV-visible absorption spectrum of the biosynthesized AgNPs is shown in Figure 2a, and the corresponding time-dependent change in absorbance at 425 nm due to microwave exposure is shown in Figure 2b. The maximum intensity was obtained at an MW- exposure (60 °C) time of 3 min, indicating the completion of chemical conversion to zero-valent silver NPs [27]. However, no leveling off effect is observed at higher exposure times, which reveals further morphological or chemical changes occurring in the system. 

The formation of the AgNPs is confirmed by a change in the color over the reaction duration due to excitation of surface plasmon vibrations [27], and the optimal synthesis time was selected as 3 min for the subsequent studies, as reaction time has been identified as one of the factors affecting the yield of AgNPs. The UV-Visible absorbance was recorded after different heating time intervals from 1 min to 10 min from the initiation of the reaction. The visual (brown color) observation and UV-Vis spectra revealed that the formation of AgNPs occurred within three minutes.

### 3.2. Transmission Electron Microscopy (TEM) and Dynamic Light Scattering (DLS) Analysis

**Figure 3:**
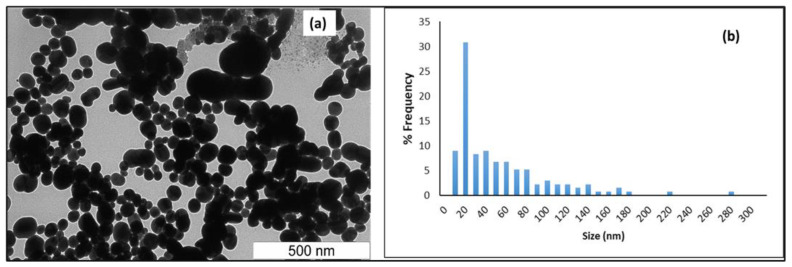
(**a**) TEM image of AgNPs (**b**) Histogram of Image J excel result of synthesized Ag nanoparticles.

**Figure 4:**
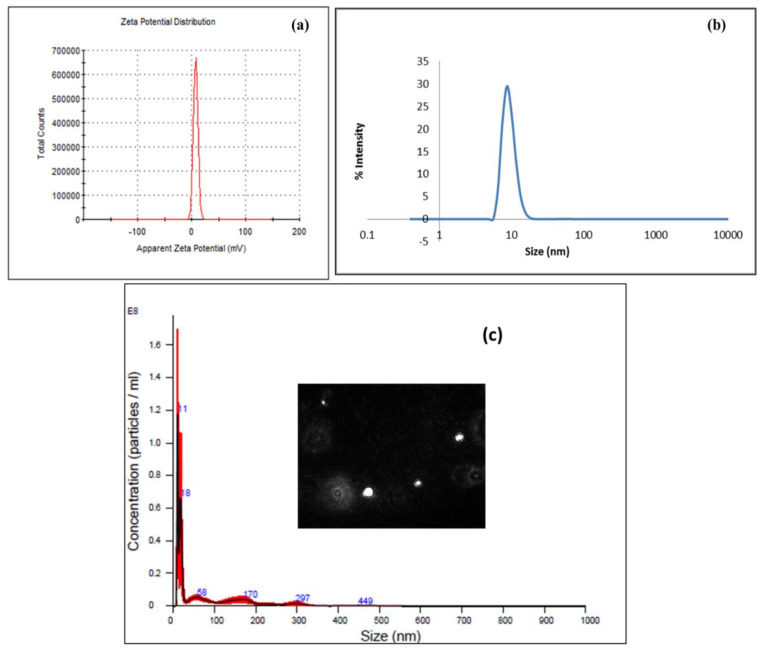
(**a**) Zeta potential of AgNPs by DLS, (**b**) Histogram showing the calculated particle size of AgNPs by DLS (**c**) Nanoparticle tracking analysis showing the Brownian motion of AgNPs, and that most of the particles are <30 nm.

The size, shape, and morphology of the AgNPs synthesized were determined through TEM, and the resulting image is shown in Figure 3a. The heterogeneity in the size of the NPs is visible and reveals that NPs are well dispersed and spherical (shape), while some have irregular structures. However, image J analysis indicated that greater than 80% of the particles had a size (diameter) in the range of 15–20 nm. The image analysis also indicates negligible agglomeration, which is expected, considering the variety of chemical species present in leaf extract that act to stabilize nano-sized metal clusters, as shown in Figure 3b. The DLS and Nanoparticle tracking analysis (NTA) data confirm the particle size and dispersion characteristics (Figure 4b,c) and the particles are found to be slightly positively charged (Figure 4a).

### 3.3. X-ray Diffraction (XRD) Analysis of AgNPs

**Figure 5:**
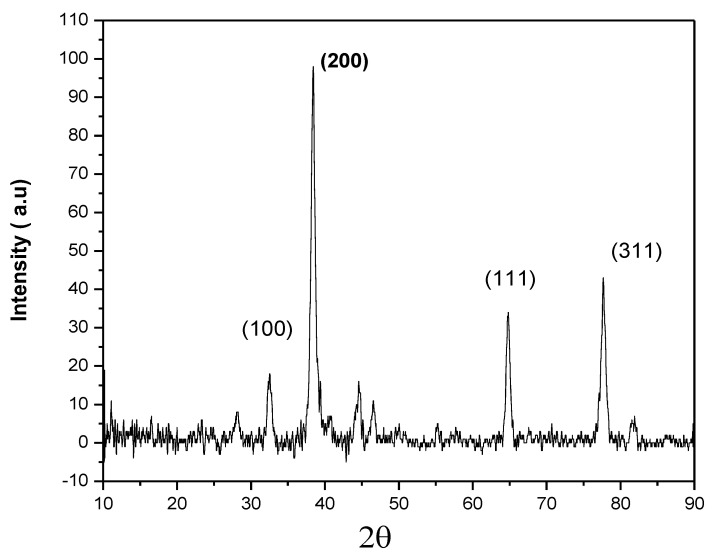
XRD analysis of the free AgNPs.

Figure 5 depicts the XRD pattern of the synthesized AgNPs. Typical peaks at 32.50°, 38.36°, 44.56°, 64.81°, and 77.66° were assigned to (100), (200), (220), (111), and (311) planes and compared with the standard powder diffraction card of JCPDS (File No. 04-0783) that confirmed the presence of silver ions and the crystalline nature of the synthesized AgNPs. No peaks were observed that would correspond to the presence of impurities. XRD revealed the monoclinic structure of the synthesized AgNPs.

### 3.4. Atomic Force Microscopy (AFM) Analysis of AgNPs Formation 

**Figure 6:**
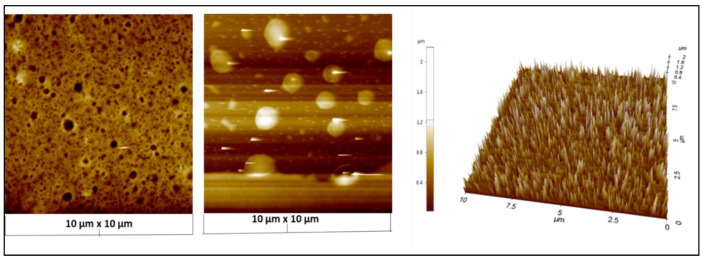
AFM analysis of AgNPs and 3D magnification over 10 μm × 10 μm.

Figure 6 depicts a 3D AFM image of the AgNPs, and the brighter spots confirm the location of the NPs due to their high modulus as compared to the mica surface. Further, it is confirmed that the AgNPs are of uniform size and are spherical. An XE-100 Advanced Scanning Probe Microscope was used to study the surface roughness of the NPs before and after embedding them into the cellulose acetate membrane. There were no signs of aggregation with the naked eye, indicating that NPs were well dispersed throughout the polymeric matrix.

### 3.5. Incorporation of Ag Nanoparticles into the Cellulose Acetate Membrane

**Figure 7:**
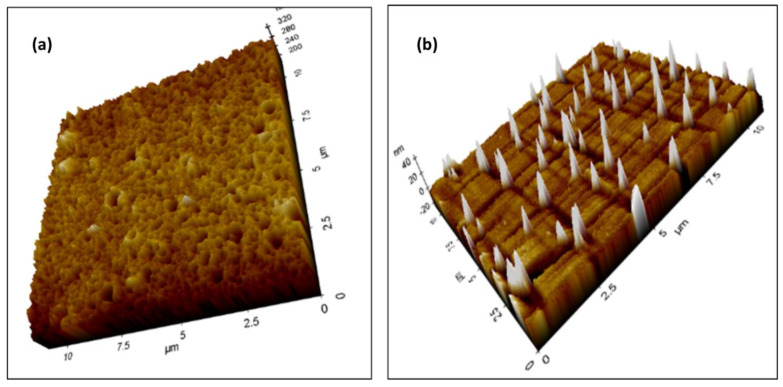
3D images via AFM analysis (**a**) Illustration of the porosity and (**b**) dispersion of AgNPs throughout the polymeric matrix, scanning over a 10 μm × 10 μm area.

To assess sample uniformity, topography and other surface properties were studied over a larger area at various locations. Smaller sections were scanned at higher resolution. The surface roughness and porosity of the cellulose acetate membrane were found to be altered by incorporation of the AgNPs—pores of micrometer size were formed after incorporation of NPs into the membrane, as represented in the 3D image of AFM (Figure 7a), while the surface roughness of the membrane was not changed by the modification with AgNPs.

### 3.6. Removal of Cypermethrin from Water Using AgNPs and Cellulose Acetate Membrane Incorporated with AgNPs

**Figure 8:**
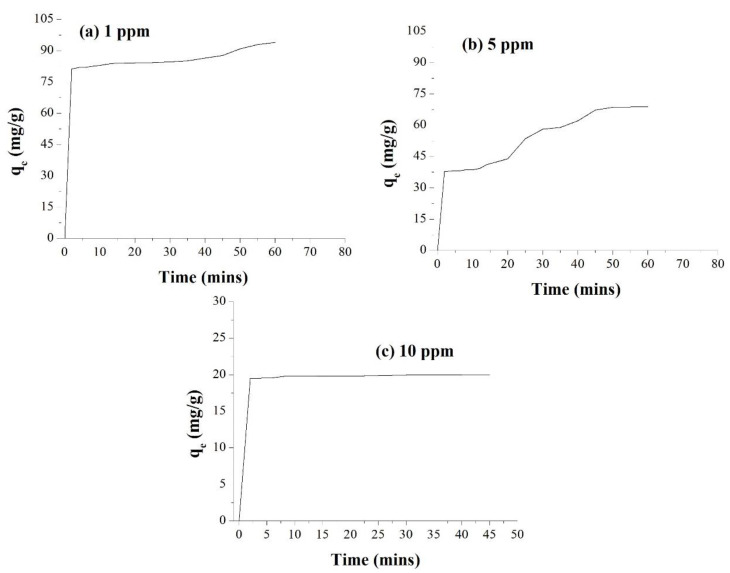
% Adsorption of the insecticide CP by free AgNPs at different initial AgNPs concentrations.

Both CP and CA belong to the well-known nereistoxin insecticidal class of pesticides, which act by blocking the nicotinic acetylcholine receptor. The interaction of these pesticides with NPs results in binding of the pesticide to the reactive surface of the NMs, potentially displacing some of the mint extract components, or binding to them [51,52,53]. The adsorption of pesticides on AgNPs was first followed spectrophotometrically, while NPs can be spread homogeneously on the substrate, so the fraction of surface atoms becomes large. At a constant concentration of 0.1 g AgNPs/100 mL of the CP solution, and thus a constant surface area for binding, the removal of pesticide at room temperature was observed. Increasing the concentration of CP from 1 ppm to 5 ppm and 10 ppm resulted in decreased CP removal efficiency due to saturation of the binding sites. The decreased percent removal with an increase can be explained by the fact that the active sites of AgNPs become saturated above a certain concentration. The absorbance intensity of the solution decreases as the concentration of pesticide decreases because of binding to the AgNPs, which removes the pesticide from the solution; at higher initial CP concentrations (5 ppm, and 10 ppm) a very rapid initial decrease is observed within the first 10 min due to the availability of more active sites, followed by a slower removal phase over the subsequent 20–60 min, as shown in Figure 8.

The AgNPs showed a promising result by absorbing 93.9% of CP from a 1 ppm solution within 60 min. At a higher concentration of the insecticidal solution and a constant AgNPs concentration, the AgNPs absorbed 68.8% of the 5 ppm solution with the first 60 min, and 20.02% of the 10 ppm solution in less than 3 min. It was found that the AgNPs showed a promising result in the removal of insecticide. As shown in Figure 8, adsorption started rapidly and reached a plateau within 20 min, which could be related to the availability of more binding sites at the beginning, which became saturated with time, and second, a gradual decrease in the concentration gradient between the bulk solution and adsorbent was evident. Similarly, with unchanged adsorbent mass and volume, the ratio of active sites to the ions of CP became lower at a higher initial concentration, which led to a decrease in the percentage of removal.

To assess the impact of stabilizing the AgNPs into a cellulose acetate membrane, a small portion (2 cm) of polymeric membrane incorporated with 0.1 g of AgNPs was cut and immersed into the samples containing the CP insecticide. After immersion of the membrane, 2 mL of the solution was checked by a UV/Visible spectrophotometer. The AgNPs polymeric membrane gave an excellent result, by adsorbing 99.8% of 1 ppm insecticide (CP) solution, due to the availability of more surface area, within the time duration of only 20 min. While in the case of 5 ppm and 10 ppm, the membrane took 30 to 35 min for the adsorption of essentially all available CP. The same studies were carried out with the blank membrane prepared without incorporation of AgNPs, whereby it was observed that within the first five minutes the blank membrane reaches a constant absorption level with no change in the value after the initial 5 min.

The results shown in Table 2 demonstrate that the AgNPs individually adsorbed 93.9% of CP from the 1 ppm water sample within 60 min while AgNPs showed better adsorption after fabrication into the polymeric membrane. After fabrication into the polymeric membrane, not only was the time of adsorption reduced but also effectiveness increased as determined by the increased % adsorption of CP.

### 3.7. Removal of CA and PQ by Using AgNPs and Membrane Incorporated with AgNPs

**Figure 9:**
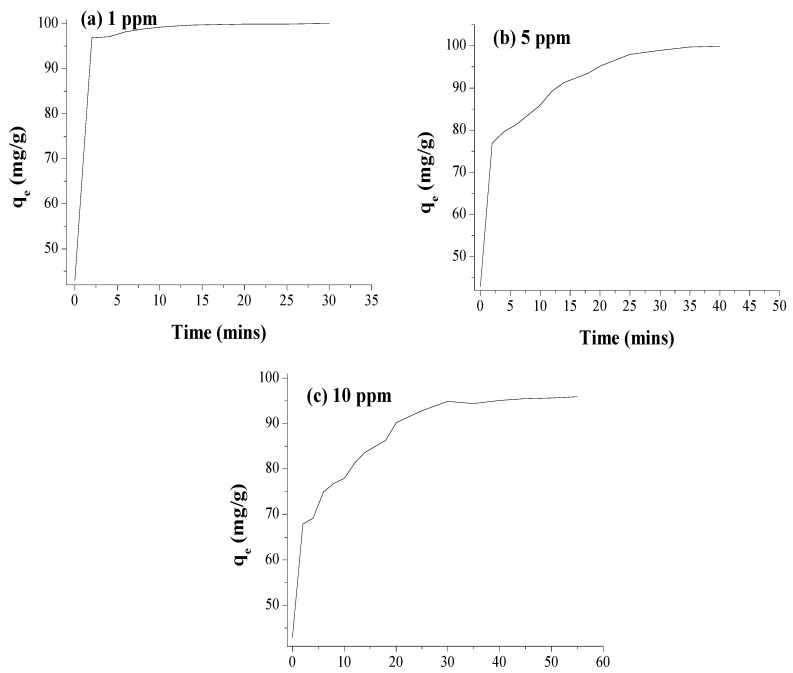
% Adsorption of CA from water by free AgNPs at different initial AgNPs concentrations.

Figure 9 represents the time-dependent removal of CA at different concentrations at constant NP concentration. It is concluded that the time taken for removal increases as the pesticide concentration increases, due to the limited number of sites for absorption, and that the number of active sites determines the rate of pesticide removal.

By applying the kinetics equations, it is concluded that 100% of CA insecticide was adsorbed by the AgNPs incorporated membrane within 60 min. In the case of CA, the membrane was found to be effective and efficient enough to remove 99.9% of insecticide of different concentrations from the water sample within 20–45 min, as shown in Table 3. 

**Figure 10:**
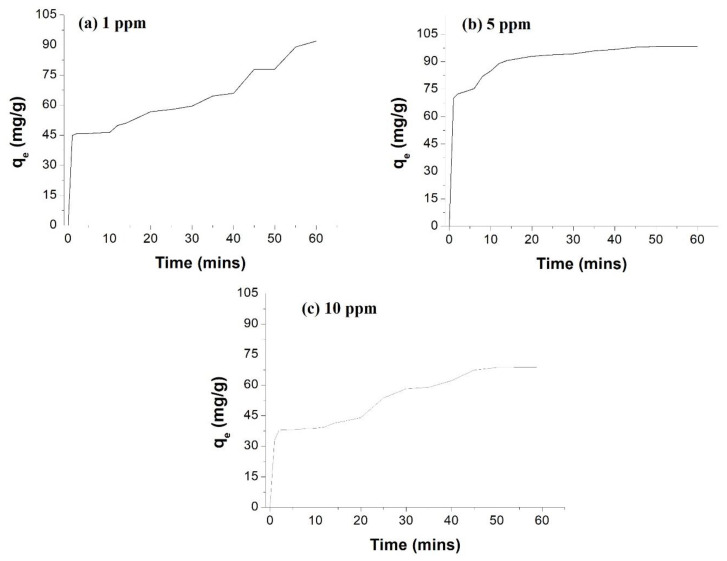
% Adsorption of PQ herbicide from water by free AgNPs at different initial AgNPs concentrations.

A similar study was conducted for the removal of PQ herbicide from the water sample using AgNPs and a fabricated polymeric membrane. The synthesized AgNPs adsorbed 91.9% to 98.9% PQ herbicide with the same time duration (60 min), as confirmed in Figure 10. Again, the AgNPs incorporated membrane showed its efficiency by minimizing the time of adsorption by adsorbing 100% of 1 ppm PQ from water in no more than 20 min, as shown in Table 4. The blank membrane has shown no prominent role in the absorption efficiency for the respective herbicide, reaching saturation at a low level of adsorption after five minutes of exposure. 

## 4. Discussion

AgNPs are one of the mostly intensively explored nanostructures due to their unique physicochemical properties and widespread biomedical applications as catalysts and as antimicrobial agents [47]. Recently, green approaches for the synthesis of NPs are becoming increasingly accepted due to the availability of a vast array of biological resources, the ability to produce high density of NPs, the stability of the resulting NPs, the decreased time requirement, and the ready dispersibility of the prepared NPs [48]. Metallic NPs have a unique catalytic activity in the degradation of halocarbons and other organic molecules, as well as inorganic contaminants. Membranes are considered to play a pivotal role in these degradative processes by giving support for the NPs dispersion [49]. NPs incorporation into membranes has the additional advantage of decreasing chemical usage for membrane cleaning and fabrication through the antimicrobial properties of the AgNPs, thus reducing energy needs compared to conventional methods [50,51,52,53]. Yorseng et al. (2020) demonstrated that hydrothermally generated Ag NPs can be successfully utilized as a cost-effective antibacterial household cleaner and as an antibacterial filler in polymeric matrices to produce antibacterial nanocomposites [54]. NPs offer high surface area and thus numerous active sites for contaminant adsorption, while NPs incorporated into membranes show interesting properties for the removal of various trace amounts of pollutants from water by combining adsorption and filtration due to their high surface area and porosity. From an aqueous solution, the adsorbent is captured via chemical binding due to the presence of surface functional groups on the NPs, whereas physical adsorption due to van der Waals forces (porosity and high surface area of NPs) or electrostatic attraction (negatively charged nanoparticles attract positively charged metal ions) also play an important role in the binding process.

Some important information on the properties, applications, and hazards of CP, PQ, and CA is provided in Table 1. CA is a mutagenic agent, whereas CP and PQ are possible human carcinogens. Besides ingestion, they can penetrate the body through the skin and via inhalation. They attack the respiratory and central nervous systems. The bioaccumulation of CP or the logarithm of the octanol-water (ratio of chemical concentration in octanol, C_o_) partition coefficient (log K_ow_) for CP exceeds +3.0, and inherent toxicity and persistence values are also high. On the other hand, the bioaccumulation of PQ is unlikely owing to the negative value of its octanol-water partition coefficient, suggesting that it prefers aquatic environments to fatty membranes. 

The resulting AgNPs show a broad absorption band in the visible region of the electromagnetic spectrum, as these metal NPs show an intense color at the nanoscale which is absent in their bulk counterparts, as well as at their atomic level. The main reason behind this phenomenon is attributed to the collective oscillations of the free conductive electrons that are induced by an interaction with the electromagnetic field, known as localized surface plasmonic resonance (SPR). The presence of this band is thus used to confirm the formation of NPs and provides information on their size and shape based on the wavelength of the peak maximum and the peak shape. AgNPs SPR, and their non-bleaching properties, show significant potential for single-molecule labeling-based biological assays and near-field optical microscopic applications as a result of augmented signal output [32]. Particle size is an important property that influences the uptake and effects of NPs. Smaller particles display better antimicrobial and cytotoxic activity, as the specific surface area increases with decreasing particle size, with a greater proportion of the atoms being displayed on the surface for smaller particles. This also implies that for the same mass of NPs, biological interactions and toxicity are more dependent on particle number and surface area than on the particle mass. Additionally, the properties of AgNPs can be enhanced by NPs functionalization by different coatings and fabrication routes that influence the surface charge, solubility, and hydrophobicity, and thus the NPs binding capacity for other organic and inorganic constituents of their surroundings.

The number of pesticide molecules adsorbed by AgNPs and AgNPs incorporated into the cellulose acetate membrane was determined using the equilibrium adsorption equation. The results indicate that the main adsorption occurred as a monolayer or through the fixed number of identified active sites on the AgNPs. As reported earlier, AgNPs are an effective biocidal agent and are broadly employed for the fabrication of antifouling membranes due to their strong inhibitory and biocidal performance against various microorganisms and low toxicity toward mammalian cells for long-term applications. Both AgNPs and released Ag^+^ ions can kill bacteria and prevent biofilm formation on the membrane surface; however, the AgNPs also act as a controllable source of Ag ions, demonstrating more lasting effects than Ag ions directly incorporated into the membranes. Given that the WHO have highlighted that that too high concentrations of Ag^+^ ions are harmful to human beings [55], use of the AgNP form facilitates effective slow release while maintaining the level of Ag ions below the harmful effects threshold. The AgNPs can be fixed into the membrane using a range of approaches such as surface grafting, physical blending, surface coating, and characterization of the stability of AgNPs incorporated into the membrane is important to ensure the long-term anti-biofouling property of the membrane but also to guarantee a low Ag concentration in the aqueous solution.

## 5. Conclusions

AgNPs were successfully synthesized from *Mentha piperita* extract and proved to be highly effective in removing pesticides from aqueous solutions. The stability of the AgNPs was confirmed by UV-Visible spectrophotometry while the SPR band was found at 425 nm. TEM images verified the formation of well-dispersed and spherical-shaped AgNPs with a mean size of 20 nm. AFM demonstrated an even distribution of AgNPs incorporation in the cellulose acetate membrane and no agglomeration was observed. The adsorption of pesticides on AgNPs and AgNPs incorporated membrane was investigated in an aqueous solution at room temperature. 0.1 g adsorbents (AgNPs) were found to be efficient and effective at lower pesticide concentrations, by removing the the maximum amount of pesticides from the water sample within 60 min, due to the presence of an optimal amount of active sites. AgNPs were successfully incorporated into the polymeric membrane and applied for the adsorption and were also shown to successfully remove the CP, CA, and PQ from the aqueous solution with an efficiency of more than 90%. Time and concentration were the main determinants of adsorption efficiency when keeping the adsorbent dosage constant. The AgNPs’ incorporated membrane provides an efficient approach to completely remove 97.6–100% of the pesticides in less than 30 min. The membrane showed promising results at lower concentrations of pesticides, whereas at higher pesticide concentrations (5 ppm and 10 ppm), more saturation of active sites was observed which could potentially be overcome with higher NP loading. Hence, it can be concluded that green-synthesized AgNPs can be utilized for the effective adsorption of pesticides from contaminated water. The nano-adsorbent has been found to be very efficient and may be employed for the degradation/removal of other toxic pollutants in the future. 

## Figures and Tables

**Table 1:** The various properties and harmful effects of the pesticides utilized in this study.

Herbicide/ Insecticide	Use	Approval	Toxicity Threshold		Log *K_ow_*	LD_50_
			Human	Marine life		
Cypermethrin (CP)	Insecticide for cotton, fruits, and vegetables, Commercial and residential settings	EPA, US for restricted control of insects	Long-term, High	Extra high for fish	>4.5 (bioaccumulation expected)	57,500 μg/kg (oral, rat)
Cartap hydrochloride (CA)	Targets chewing and sucking insects on many crops, including rice.	1967 Japan, USChewing and sucking pests	Long-term, very low	Long-term, very low	0.0	250–340 mg/kg (oral, rat)
Paraquat (PQ)	Herbicide for weeds and grasses in agricultural & non-agricultural areas	EPA, US restricted use of pesticide	Long-term, high	Not available	−4.0 (unlikely to bio accumulate)	110–150 mg/kg (rats)

**Table 2:** % Adsorption of Cypermethrin (CP) insecticide by free AgNPs and AgNPs incorporated into a cellulose membrane, as a function of the initial insecticide concentration.

Adsorption of CP by 0.1 g AgNPs				Adsorption of CP by 0.1 g AgNPs Incorporated into the Cellulose Membrane			Adsorption with the Membrane (without AgNPs)
	1 ppm (CP)	5 ppm (CP)	10 ppm (CP)	1 ppm (CP)	5 ppm (CP)	10 ppm (CP)	
% Adsorption	93.9	68.8	20.02	99.8	97.9	95.1	0.6
Adsorption Time (min)	60	60	60	20	30	35	60

**Table 3:** % Adsorption of Cartap (CA) by free AgNPs and AgNPs incorporated into a cellulose acetate membrane, at different initial concentrations of the insecticide.

Adsorption by 0.1 g AgNPs				Adsorption of CP by 0.1 g AgNPs Incorporated into the Cellulose Membrane			Adsorption with the Membrane (without AgNPs)
	1 ppm (CA)	5 ppm (CA)	10 ppm (CA)	1 ppm (CA)	5 ppm (CA)	10 ppm (CA)	
% Adsorption	100	79.1	66	100	91.2	89.7	0.5
Adsorption Time (min)	35	60	60	20	35	45	60

**Table 4:** % Adsorption of Paraquat (PQ) by free AgNPs and AgNPs incorporated into the cellulose acetate membrane with different initial insecticide concentrations.

Adsorption by 0.1 g AgNPs				Adsorption by 0.1 g AgNPs Incorporated into the Cellulose Membrane			Adsorption with the Membrane (without AgNPs)
	1 ppm (PQ)	5 ppm (PQ)	10 ppm (PQ)	1 ppm (PQ)	5 ppm (PQ)	10 ppm (PQ)	
% Adsorption	93.5	51.3	73.2	100	96.5	92.7	0.7
Adsorption Time (min)	60	60	60	25	30	30	60

## Data Availability

The data presented in this study are available on request from the corresponding author.
